# Exploratory Evaluation of Hepatic Venous Pressure Gradient, Indocyanine Green Retention, and Liver Stiffness in Predicting Short-Term Outcomes After Liver Resection in Patients with Cirrhosis: A Multicenter Prospective Study

**DOI:** 10.3390/cancers18142300

**Published:** 2026-07-17

**Authors:** Gianluca Cassese, Mariano Cesare Giglio, Nadia Russolillo, Francesco Razionale, Andrea Laurenzi, Boram Lee, Gianluca Rompianesi, Filomena Morisco, Ho-Seong Han, Matteo Cescon, Felice Giuliante, Alessandro Ferrero, Roberto Montalti, Isabelle Colle, Roberto Ivan Troisi

**Affiliations:** 1 Division of Minimally Invasive and Robotic HPB Surgery, Transplantation Center, Federico II University Hospital, 80131 Naples, Italy; 2 Department of Health Sciences, University of Eastern Piedmont “A. Avogadro”, 15121 Alessandria, Italy; 3 Department of Clinical Medicine and Surgery, University of Naples Federico II, 80131 Naples, Italy; 4 Department of General and Oncological Surgery, Mauriziano Hospital “Umberto I”, 10100 Turin, Italy; 5 Division of Hepatobiliary Surgery, Fondazione Policlinico Universitario Agostino Gemelli IRCCS, 00168 Rome, Italy; 6 Hepatobiliary Surgery and Transplantation Unit, Azienda Ospedaliero-Universitaria di Bologna IRCCS, 40138 Bologna, Italy; 7 Department of Surgery, Division of HPB Surgery, Seoul National University Bundang Hospital, Seongnam 13620, Republic of Korea; 8 Department of Clinical Medicine and Surgery, Diseases of the Liver and Biliary System Unit, University of Naples Federico II, 80131 Naples, Italy; 9 Department of Public Health, University of Naples Federico II, 80131 Naples, Italy; 10 Department of Gastroenterology and Hepatology, A-Zorg Campus Merestraat, 9300 Aalst, Belgium

**Keywords:** HVPG, hepatocellular carcinoma, portal hypertension, liver stiffness, indocyanine green

## Abstract

**Introduction:** The correct risk stratification is the key to achieving a low postoperative mortality after liver resection for HCC, including the evaluation of liver function and the presence of portal hypertension. The aim of this study was to evaluate the accuracy of Hepatic Venous-Portal pressure Gradients (HVPG), Indocyanine Green retention test (ICG-R15) and Liver Stiffness (LSM) in predicting the risk of post-hepatectomy liver failure and major postoperative complications in selected cirrhotic patients. **Methods:** A multicenter prospective study was designed, including consecutive cirrhotic patients undergoing liver resection for HCC. The predictive ability of HVPG, LSM and ICG-R15 was tested by evaluating the Area Under the ROC Curves. **Results:** Ninety-eight cirrhotic patients undergoing liver resection for HCC were included. Twelve patients (12.2%) developed at least one of the complications included within the composite endpoint. Three patients (3%) developed post-hepatectomy liver failure (PHLF) (one graded A and two graded C according to the International Study Group of Liver Surgery), five patients (5.1%) developed large ascites, five patients (5.1%) developed severe postoperative complications, two patients (2%) died during the hospital stay (PHLF). HVPG showed the higher AUC (0.778, 95% CI 0.641- 0.898; *p* < 0.001), followed by LSM (0.669, 95% CI 0.471–0.847; *p* = 0.097) and ICG-R15 (0.593, 95% CI 0.378–0.788; *p* = 0.615). **Conclusions:** In our cohort of selected HCC patients undergoing elective surgery, the predictive ability of HVPG on developing a composite endpoint including PHLF, in-hospital mortality, large postoperative ascites and major postoperative complications is higher than LSM and ICG-R15.

## Simple Summary

This multicenter prospective study evaluated the role of HVPG, LSM, and ICG-R15 in predicting postoperative PHLF risk in cirrhotic patients undergoing liver resection for HCC. Among 98 patients, 12.2% developed major complications, including three (3%) liver failures and two deaths (2%). HVPG showed the best predictive accuracy compared to LSM and ICG-R15. These results suggest that HVPG is the most reliable tool for identifying patients at higher risk of postoperative complications and liver failure after surgery.

## 1. Introduction

Liver malignancies account for the fifth most common cancer worldwide and the fourth most common cause of cancer-related death [1]. With an estimated incidence between 500,000 and 1 million per year, hepatocellular carcinoma (HCC) represents approximately 90% of liver tumors, associated with a poor prognosis [2]. Nonetheless, when it is diagnosed in early stages, 5-year overall survival (OS) reaches 50–70%, thanks to advances in both surgical and medical fields [3] HCC develops in the setting of underlying chronic liver disease in more than 90% of cases, more commonly due to viral, metabolic, or alcohol-related etiologies, and most frequently in the presence of liver cirrhosis [4]. In this context, the risks associated with the underlying liver disease add to the morbidity and mortality related to the tumor itself [5].

Surgery represents the most effective potentially curative option for HCC, as it has been shown to be safe and effective when appropriate patient selection and state-of-the-art surgical techniques are applied [6]. However, liver resection is a technically demanding procedure, associated with significant postoperative morbidity and mortality, particularly in cirrhotic patients [5,7]. Therefore, accurate risk stratification and tailored patient selection are crucial in the management of HCC, allowing postoperative mortality rates below 1% in high-volume specialized centers [8]. Currently, European guidelines for the treatment of HCC are based on the Barcelona Clinic Liver Cancer (BCLC) staging system [9]. According to this staging system, patients are classified based on Child–Turcotte–Pugh (CTP) class, number and size of lesions, and performance status (PS) of the patient [10]. Portal hypertension (PHT) has traditionally been considered a major adverse risk factor and a relative contraindication to resection in many guidelines, although selected patients may safely undergo resection in expert centers [11]. However, advances in surgical techniques and patient management have led many centers to overcome the limitations imposed by the BCLC system, highlighting the need for more refined selection criteria [12]. In this regard, Ishizawa et al. from the University of Tokyo reported a large case series of patients undergoing hepatic resection despite PHT, with favorable results [13]. In their study, multivariable analysis showed that PHT was not an independent predictor of OS, whereas a CTP-B Class was significantly associated with worse outcomes. Subsequent studies have further demonstrated how the results can vary within the same CTP-B class, based on additional factors, such as the use of minimally invasive access, as shown in recent multicenter analyses [14].

Within the preoperative assessment of patients undergoing liver resection, a key role is played by the volumetric calculation of the future liver remnant (FLR), as well as by the evaluation of liver functional reserves. The latter can be commonly evaluated by the blood clearance of Indocyanine Green (ICG), and specifically by its retention rate after 15′ from injection (ICG-R15) [15,16]. In Eastern countries, ICG-R15 is routinely used to guide surgical decision-making in cirrhotic patients, particularly within the framework of the Makuuchi criteria, which integrate FLR and the extent of resection [17]. However, there is high intra- and inter-individual variability for ICG measurements, and its reliability is impaired in case of hyperbilirubinemia [18].

Almost all the studies in the literature investigating the prognostic role of PHT in patients undergoing liver surgery are based on retrospective data and rely on indirect evaluation of the presence of PHT rather than direct measurement of hepatic venous portal gradients (HVPG). Indeed, HVPG represents the gold standard for assessing portal pressure, but it is an invasive procedure, defined by the difference between hepatic vein free pressure and hepatic vein wedge pressure, obtained via selective catheterization [19]. Indirect surrogates such as splenomegaly associated with low platelets (<100,000/cm^3^) or the presence of esophageal varices are the most commonly used [20]. More recently, non-invasive assessment of portal hypertension through liver stiffness measurement (LSM) using transient elastography (FibroScan^®^) has gained increasing acceptance. Current guidelines recommend avoiding screening with gastroscopy in patients with liver stiffness (LSM) < 20 kPa and platelet count > 150,000 [19]. Nonetheless, LSM has been shown to be associated with postoperative complications after liver resections [21].

The aim of this study was to evaluate the performance of HVPG (direct method, measured invasively through venous catheterization), ICG-R15 (non-invasive method, measured through Limon device) and LSM (non-invasive method, measured through elastography) in predicting the risk of post-operative liver failure and hepatic decompensation.

## 2. Methods

### 2.1. Study Design

This is a multicenter prospective non-interventional study involving the following participating centers: Naples, Rome, Bologna, Turin and Seoul. Consecutive cirrhotic patients with an indication for surgical resection for HCC were included. Patients were selected for surgery based on standard criteria according to current guidelines, i.e., remaining liver volume (≥50% of total liver volume), PS 0 and Child class A or B, after being discussed in the institutional multidisciplinary team (MDT). The exclusion criteria were the following: patients undergoing emergency surgery; patients unable to understand or to sign an informed consent.

Patients eligible for enrollment were adequately informed, and informed consent was obtained. The study was conducted according to Helsinki Declaration, and ethical approval was obtained from the Institutional Review Board of each center (Prot. 368/20).

### 2.2. Preoperative Workup

The HVPG was given by the difference between the wedge hepatic venous pressure (WHVP) and the free hepatic venous pressure (FHVP). FHVP was measured by holding the catheter tip free in the hepatic vein lumen, 2–4 cm from its opening into the inferior vena cava; its value should be similar to that of the inferior vena cava pressure. WHVP was measured by occluding the hepatic vein, and the catheter was “stuck” in a small branch of the hepatic vein by inflating a balloon at the tip of the catheter. Adequate occlusion of the hepatic vein was confirmed by slowly injecting contrast medium into the vein without observing its reflux or its passage through communications with other hepatic veins [22].

Hepatic vein catheterization was performed under local anesthesia in combination with non-invasive monitoring of vital signs (electrocardiography, blood pressure, and pulse oximetry). Since currently none of the involved centers have clinically significant portal hypertension (CSPH) as an absolute contraindication to surgery in their institutional protocols, HVPG did not influence surgeons’ decisions. Subsequently, no patients have been excluded after HVPG measurement.

Indocyanine green (ICG) clearance was measured using pulse spectrometry. Patients received 0.25 mg/kg of ICG intravenously injected the day before liver resection. Plasma disappearance rate (PDR) and ICG retention rate at 15 min (ICG-R15) were measured by pulse spectrometry with a LiMON device (Pulsion Medical Systems, Munich, Germany). Liver stiffness (LSM) was assessed by Liver Elastography under ultrasound guidance. LSM values were obtained according to the European recommendations of the Federation of Societies for Ultrasound in Medicine and Biology (EFSUMB) [23]. For each patient, the LSM value was considered adequate if the success rate was >60% and the interquartile range (IQR) was <30% of the median value.

### 2.3. Patients’ Management

The patients followed the normal therapeutic management of any other patient undergoing liver surgery. After the surgery, patients were managed in mid-care unit for 24 h in case of no postoperative complications, where they received careful management of fluid therapy, pain therapy, monitoring of vital parameters and drainage. Upon return to the surgical ward, a fast-track surgery protocol was applied where possible [24]. Fluid management was carried out following an equal or slightly negative daily balance [25]. Antibiotic prophylaxis was carried out according to current recommendations, of a short-term nature [26]. Pain therapy was conducted according to center’s policy. Upon returning to the ward, oral feeding and mobilization were early re-introduced. Patients’ clinical conditions and laboratory tests were evaluated daily, as well as the development of any complications.

After discharge, patients were followed as outpatient after 3 weeks, 3 months, 6 months and 12 months. A clinical evaluation was carried out to investigate the presence of surgical complications, together with a biochemical evaluation of liver function. The state of liver function was then assessed according to the CTP and MELD scores. Any clinical manifestations of cirrhotic decompensation were carefully investigated: hepatic encephalopathy, acute kidney injury, jaundice, ascites.

### 2.4. Definitions

Post-hepatectomy liver failure (PHLF) was defined according to the International Study Group Liver Surgery (ISGLS) criteria [26]. Clinically significant portal hypertension (CSPH) was defined as HVPG > 10 mmHg. Ascites in the postoperative period was defined as drainage production greater than 10/mL/kg/24 h [27]. Postoperative ascites was considered clinically significant (large ascites) when drainage output exceeded 500 mL/day for at least 3 consecutive days, in line with the previous surgical literature.

Hepatic encephalopathy was defined clinically based on two types of symptoms, impaired mental status and impaired neuromotor function, as defined by the West Haven Criteria (WHC) [28]. Postoperative complications were defined according to the Clavien–Dindo classification [29].

### 2.5. Statistical Analysis

Clinical, instrumental, and laboratory data were collected at the beginning of the study (at the time of hospitalization and during the intervention) and at 3, 6, 9 and 12 months following surgery. Patients were monitored in order to record the clinical course, the development of post-surgical complications and mortality. Any complications that developed during hospitalization, the values of the liver function and cholestasis, the duration of hospitalization, and any transfusion of blood products were recorded.

The primary endpoint was the comparison of the predictive accuracy (evaluated as Area Under the Curve of the ROC curve—AUC) of HVPG, ICG-R15 and LSM for a composite endpoint defined as the development of PHLF, severe postoperative ascites, in-hospital mortality, severe postoperative complications (defined as more than 3A according to Clavien–Dindo classification). Secondary endpoints were 3-month morbidity and mortality outcomes. Continuous data were expressed as mean and standard deviation (SD) or median and interquartile range (IQR) depending on whether they had a normal distribution, and comparisons between groups were performed using Student’s *t* test or Wilcoxon test, depending on the distribution of the variable.

Categorical data were expressed as frequencies and associated percentages. Comparisons between groups were performed using Pearson’s Chi Square test. The presence of a linear correlation between the variables was verified by linear regression. The accuracy of a continuous variable in predicting a categorical outcome was assessed using Receiver Operating Characteristic (ROC) curves. In particular, the value of the Area Under the Curve (AUC), reported together with the 95% confidence interval, was used as an index of predictive accuracy. Sensitivity and specificity were calculated for different cut-off values of each continuous variable. Youden’s *J* test was performed to evaluate the performance of a positive score on the composite endpoint occurrence. *p* values ≤ 0.05 were considered statistically significant. Statistical analysis was conducted using SPSS software (version 28.0). Pairwise comparisons between AUCs were performed using paired DeLong tests, accounting for the fact that the ROC curves were derived from the same patients. Differences between AUCs were reported with corresponding 95% confidence intervals and *p*-values. All analyses were performed in R using the pROC package (version 4.5.3). To account for potential confounding related to surgical extent and approach, we performed predefined subgroup analyses. Patients were stratified according to extent of resection (minor vs. major resection, with major defined as resection of ≥3 segments); surgical approach (laparoscopic vs. open surgery); liver function (Child–Pugh A-only subgroup analysis). Given the limited number of patients in some subgroups, these analyses were considered exploratory.

## 3. Results

### 3.1. Patients’ and Tumors’ Characteristics

From January 2022 until December 2023, ninety-eight cirrhotic patients undergoing liver resection were prospectively enrolled. Median age was 72 ± 11 years, with a median BMI of 26 ± 5.3, and 83.6% of the sample were male patients. Ninety-five patients (97%) out of ninety-eight were classified as CTP A, while 3/98 (3%) patients had a Child B-7 cirrhosis. According to HCC staging, 10 patients were staged as BCLC-0, 64 patients as BCLC-A and 24 as BCLC-B.

The median HVPG value was 7.5 mmHg (IQR 5), while the median ICG-R15 was 9.1% (IQR 10.6) and the median LSM was 12 kPa (IQR 8.9).

A total of 52 patients (53%) underwent a segmentectomy, 34 (34%) patients underwent an atypical resection, 8 (8.0%) underwent a sectionectomy, and 3 left and 2 right hepatectomies (5–all laparoscopic). The majority of patients received a minimally invasive access approach: n = 72 (73.4%) laparoscopic and n = 8 (8.1%) robotic.

All patients and tumor characteristics are shown in Table 1.

### 3.2. Predictive Accuracy Analysis

**Figure 1:**
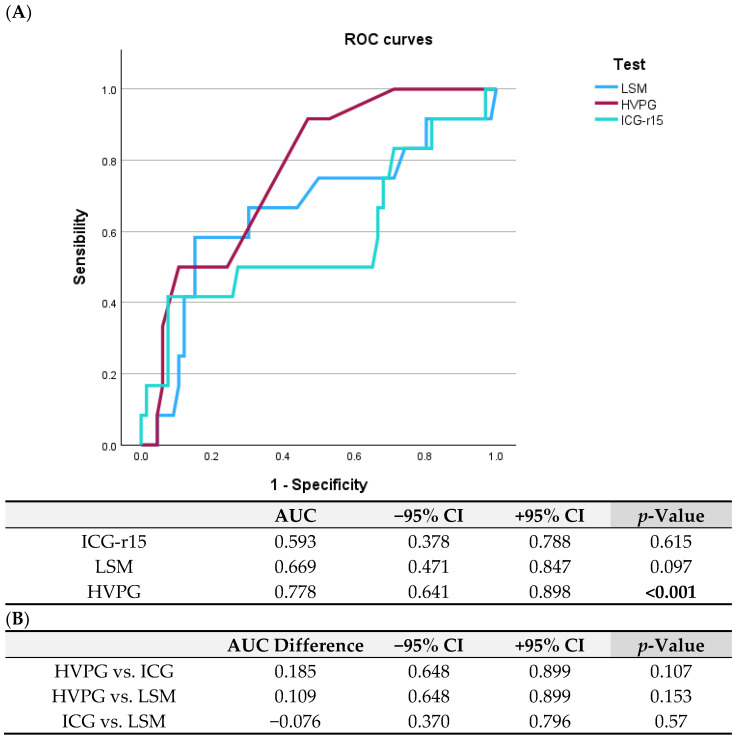
(**A**): Receiver Operating Characteristics curves for predictive ability of HVPG, ICG-R15 and LSM on the combined endpoint, with the table reporting the Area Under the Curves. In bold, significant values. ICG-r15: Indocyanine green retention test at 15 min; LSM: liver stiffness; HVPG: hepatic venous-portal pressure gradients. (**B**): Comparisons between AUC.

Twelve patients (12.2%) developed at least one of the complications included within the composite endpoint. In particular, three patients (3%) developed PHLF (one graded A and two graded C), five patients (5.1%) developed large ascites, five patients (5.1%) developed severe postoperative complications (acute kidney injury, infected ascites, respiratory failure, intraabdominal abscesses and hypovolemic shock) whereas two patients (2%) died during the hospital stay, both of them due to a grade C PHLF. HVPG showed a statistically significant AUC (0.778, 95% CI 0.641–0.898; *p* < 0.001), differently from LSM (0.669, 95% CI 0.471–0.847; *p* = 0.097) and ICG-R15 (0.593, 95% CI 0.378–0.788; *p* = 0.615) (Figure 1A). The association between HVPG and postoperative outcomes remained directionally consistent across subgroups in the exploratory subgroup analysis in the minor resection group (AUC 0.829, 95% CI 0.710–0.949; *p* < 0.01), the minimally invasive group (AUC 0.824, 95% CI 0.690–0.959; *p* < 0.01) and the Child A group (AUC 0.787, 95% CI 0.661–0.912; *p* < 0.01) (Supplementary Material Table S1).

In the pairwise comparisons of ROC, there were no statistically significant differences between HVPG and LSM (AUC difference 0.109, 95% CI 0.648–0.899; *p* = 0.153), as well as between HVPG and ICG-R15 (AUC difference 0.185, 95% CI 0.648–0.899; *p* = 0.107) (Figure 1B).

### 3.3. Portal Hypertension Analysis

Portal hypertension, defined as HVPG > 5 mmhg, was present in 47 patients (48%), while CSPH was present in 14 patients (14.2%).

Interestingly, only 35.7% of patients with CSPH had platelets lower than 100,000 per cm^3^, only 35.7% of CSPH patients showed the presence of esophageal varices, and 64.3% of CSPH patients presented splenomegaly. However, esophageal varices, low platelets, splenomegaly, the CTP class and LSM were significantly associated with CSPH, while ICG-R15 was not (Table 2).

When focusing on the outcomes, CSPH was associated with a higher rate of 90-day cirrhotic decompensation (21.4% vs. 1.2%, *p* < 0.01), and with 90-day ascites (21.4% vs. 2.5%, *p* < 0.01).

## 4. Discussion

Hepatic venous pressure gradients, LSM and ICG-R15 were prospectively evaluated in cirrhotic patients undergoing liver resection for HCC, with the aim of assessing their predictive role on a composite endpoint including PHLF and severe 90-day postoperative complications. Among these parameters, preoperative HVPG demonstrated the highest predictive accuracy compared with ICG-R15 and LSM.

PHLF is still the leading cause of mortality after liver resection in cirrhotic patients, with an incidence of up to 30% [30]. Different risk factors for PHLF have been identified, including patient-related variables, underlying liver function, and surgery-related factors [15]. While patient-related and liver-specific factors are not modifiable, surgical risk can be partially mitigated through a rigorous preoperative assessment, including evaluation of the best surgical strategy, FLR volume, and functional reserve. Liver functional reserve can be estimated through different tests, such as hepatobiliary scintigraphy or ICG-R15′. The latter has been proven to be reliable when used within the context of the Makuuchi criteria, offering the advantages of being inexpensive and easily reproducible [31,32]. Similarly, the evaluation of the underlying liver condition can rely on the measurement of LSM by transient elastography, which has been shown to correlate with severe postoperative complications [21]. Finally, another major driver for PHLF and mortality after surgery in cirrhotic patients is PHT, with HVPG measurement being the gold standard for its assessment [33]. However, HVPG is an invasive method that requires specific expertise and dedicated facilities, which may limit its applicability in some centers. Most previous studies investigating the prognostic role of HVPG in patients undergoing liver resection are retrospective. Even in our study we had to face patients’ reluctance to undergo an invasive investigation, as well as logistical constraints related to HVPG measurement. A recent meta-analysis by Kim et al. showed a correlation between LSM and HVPG, suggesting a potential role for LSM in evaluating CSPH. However, its clinical application in surgical candidates remains insufficiently explored.

To our knowledge, this is the first study to prospectively compare the prognostic role of HVPG, LSM and ICG-R15′ in predicting early postoperative outcomes in cirrhotic HCC patients undergoing liver resection.

Our results are consistent with the previous literature. A retrospective study by Stremitzer et al. reported that HVPG exceeding 5 mmHg was associated with higher rates of postoperative liver dysfunction, ascites, and longer hospital stay compared to patients with lower HVPG values [34]. Similarly, a prospective study by Boleslawsky et al. showed that elevated HVPG was associated with postoperative liver dysfunction and 90-day mortality [35]. Notably, esophageal varices, splenomegaly and thrombocytopenia (<100,000/mm^3^) were not associated with any of the endpoints. In line with these findings, none of these indirect criteria were associated with the composite endpoint in our cohort, nor were they consistently present in patients with CSPH. Several factors may account for these findings. First, patient selection and baseline characteristics likely played a role, as our cohort predominantly consisted of patients with well-preserved liver function (mainly Child-Pugh A), which may have attenuated the association between indirect signs and CSPH. Second, preoperative selection bias should be considered: surgical candidates underwent rigorous screening according to standard criteria, likely excluding patients with more advanced portal hypertension and overt clinical manifestations, thereby reducing the variability of indirect markers. Third, the surgical approach may have influenced outcomes, as the widespread use of minimally invasive techniques in our cohort may have mitigated the impact of surrogate markers of PHT in predicting short-term postoperative outcomes.

All these results may support the implementation of HVPG measurement before liver resection in selected cirrhotic HCC patients, providing an additional tool for risk stratification and patient selection. Indeed, according to current guidelines, the presence of CSPH should always be carefully evaluated, as it remains a relative contraindication to surgery [9,36]. In clinical practice, most liver centers focus on indirect markers such as esophageal varices, splenomegaly, and thrombocytopenia. However, in our cohort, these were present only in 35.7%, 35.7%, and 64.3% of patients with CSPH, respectively. Nonetheless, CSPH was associated with a higher rate of 90-day cirrhotic decompensation (21.4% vs. 1.2%, *p* < 0.01), consistent with previous studies from Bruix et colleagues [11].

To date, few studies have tried to address this lack of consensus among HPB centers regarding how PHT should be evaluated in the decision-making process [35,37,38,39]. Only a few studies have relied on direct HVPG measurement, including those by Bruix et al. and Boleslawski et al. In the former, dating back 30 years and including a cohort of 29 patients, HVPG ≥ 10 mmHg was identified as the strongest predictor of postoperative 90-day liver decompensation [11]. In an updated series of 43 patients reported by Llovet et al. in 1999, the same group confirmed that HVPG was also associated with long-term survival [40]. Similarly, the French authors focused on postoperative outcomes, concluding that a preoperative HVPG > than 10 mmHg was associated with severe postoperative complications, postoperative liver dysfunction and mortality, whereas indirect surrogates of PHT were not [35]. However, these studies were limited by small sample sizes and differences in outcomes of interest. In other available studies, PHT was defined by various indirect criteria, with the majority of them failing to demonstrate a clear association between PHT and surgical outcomes, likely due to their retrospective design and heterogeneity in endpoints [37,41]. As in the previous literature, we observed a statistically significant association between the presence of esophageal varices and HVPG values, as well as for splenomegaly [42]. However, none of these indirect markers were associated with postoperative outcomes in our study, supporting the limited accuracy of surrogate markers in this setting [13,38,39,43].

LSM has been proposed as a non-invasive surrogate of HVPG and CSPH [44,45]. A linear correlation between LSM and HVPG has been reported, although evidence in surgical populations remains limited [35,46]. While some prospective studies have identified LSM as an independent predictor of PHLF and long-term outcomes [46,47], these studies were conducted in heterogeneous populations and did not include direct comparison with HVPG. In our cohort of exclusively cirrhotic patients, LSM showed a lower predictive performance (AUC 0.669) compared with HVPG.

The Eastern approach has always been to measure ICG clearance. In a previous Japanese study including 127 patients, ICG-R15 was the best test for predicting in-hospital mortality after hepatectomy for HCC [48]. However, only a few centers in Western countries routinely measure ICG. As ICG evaluates the detoxification function of the liver while HVPG evaluates PHT and probably severity of fibrosis, both measurements may be complementary in predicting morbidity and mortality. In our study, ICG-R15 showed the lowest predictive accuracy (AUC 0.593) among the evaluated parameters. It is interesting to note that a previous study by Pind et al. reported that ICG-R15′ can be used as an indirect assessment of CSPH in compensated cirrhotic patients. However, such findings were not confirmed in our cohort, where median ICG-R15 was not significantly associated with CSPH. This may be due to the highly selected nature of our cohort. Indeed, one of the strengths of our results may be the possibility of further stratifying cirrhotic patients undergoing liver resection after careful preoperative assessment through HVPG measurement. The next step should be to identify which subgroup of these patients may benefit from this further stratification.

Although in our study HVPG showed the highest discriminatory performance among the evaluated parameters, formal pairwise comparison of ROC curves using the DeLong method did not demonstrate statistically significant differences between HVPG, LSM, and ICG-R15. These findings suggest that, while HVPG may provide superior risk stratification at a descriptive level, its predictive advantage over other non-invasive markers could not be confirmed statistically in the present cohort. This lack of statistical significance may be partly explained by the relatively limited sample size and the resulting reduced statistical power. Therefore, the observed differences in AUCs should be interpreted with caution. Larger multicenter prospective studies are needed to better clarify whether HVPG offers a true incremental predictive value over other commonly used preoperative assessment tools and to determine clinically meaningful cutoff values for risk stratification.

Despite its prospective design, this observational study has some limitations. First, it is limited by the relatively small sample size, with a low incidence of grade C PHLF events. Indeed, in light of the limited event rate, the heterogeneity of the cohort and the limited external validity, derivation of optimal cutoffs was avoided to reduce the risk of overfitting. Second, the patients were selected for surgery based on preoperative functional assessment, including ICG-R15′ clearance, carrying a clear risk of selection bias and limiting generalizability. This must be taken into account when interpreting the results, considering this study not as a clean head-to-head comparison of HVPG, LSM, and ICG-R15 in an unselected surgical population. The majority of patients underwent minimally invasive procedures, which may have contributed to the relatively low incidence of postoperative decompensation [14,49]. Moreover, the lack of long-term follow-up precludes assessment of the prognostic value of these markers beyond the early postoperative period.

Another limitation to discuss is the use of a composite endpoint that includes clinically heterogeneous events. While key components such as post-hepatectomy liver failure (PHLF) and ascites are directly related to portal hypertension and hepatic functional reserve, other complications (e.g., respiratory failure or intra-abdominal infections) may not be liver-specific per se. However, in the context of cirrhotic or high-risk patients undergoing surgery, these events are often closely interconnected. Impaired liver function and clinically significant portal hypertension can contribute to systemic inflammation, immune dysregulation, and reduced physiological resilience, thereby increasing the susceptibility to extrahepatic complications. The composite endpoint was therefore intentionally designed to capture the overall burden of severe postoperative morbidity, reflecting real-world clinical complexity rather than isolated organ-specific outcomes. Nonetheless, we acknowledge that the inclusion of non-liver-specific complications may have introduced heterogeneity in the outcome definition and could have diluted the specificity of the association with HVPG. Future studies with larger cohorts should specifically explore liver-related endpoints, such as PHLF, ascites, and liver-related mortality, to further refine the clinical applicability of HVPG in this setting. Importantly, despite this heterogeneity, the observed association between HVPG and postoperative outcomes remained clinically meaningful, supporting the hypothesis that portal hypertension reflects a global impairment of physiological reserve rather than a purely liver-specific risk factor.

Finally, HVPG measurements are a radiological procedure that has shown some risks of errors [22]. Both FHVP and WHVP values may fluctuate during the measurement, and the fluctuation may be above 1 mmHg. Furthermore, the presence of the communicating hepatic vein branch could result in an underestimated WHVP that must be reported. Nonetheless, prior to manometry, balloon occlusion angiography may influence the initial FHVP value. Indeed, the Baveno VII consensus paper produced some recommendations to reduce such errors, but it was released after the beginning of this study [19].

## 5. Conclusions

In our cohort of cirrhotic HCC patients undergoing elective surgery, the predictive ability of HVPG on developing a composite endpoint including PHLF, in-hospital mortality, large postoperative ascites and major postoperative complications was higher than LSM and ICG-R15.

Further prospective studies with dedicated designs are warranted to better define the role of HVPG in the preoperative assessment of cirrhotic patients with HCC. Such studies should aim to develop integrated predictive models combining HVPG, LSM, and ICG-R15 and to explore individualized risk stratification tools to guide surgical decision-making.

## Figures and Tables

**Table 1:** Patients’ and tumors’ characteristics.

	Total (n = 98)
Age in years, median (IQR)	72 (11)
Male gender, n (%)	82 (83.6)
BMI in kg/m^2^, median (IQR)	26 (5.3)
Etiology, n (%)	
HBV	17 (17.3)
HCV	31 (31.6)
Alcoholic	10 (10.2)
Metabolic	33 (33.7)
Viral + metabolic	7 (7.1)
Child–Turcotte–Pugh score,	
Child A, n (%)	95 (97)
Child B, n (%)	3 (3)
Recurrent disease, n (%)	20 (20.4)
Previous treatments, n (%)	24 (24.5)
Previous TACE, n (%)	4 (4.1)
Previous RFA, n (%)	10 (10.2)
Previous liver surgery, n (%)	10 (10.2)
ICG-R15, median (IQR)	9.1 (10.6)
Liver stiffness, median in KPa (IQR)	12 (8.9)
Hepatic venous-portal gradients, median in mmHg (IQR)	7.5 (5)
Esophageal varices, n (%)	13 (13.2)
Splenomegaly, n (%)	18 (18.3)
Platelets < 100 × 10^3^/mm^3^, n (%)	9 (9.1)
HCC Size, median (IQR)	33 (40)
HCC numbers, n (%)	
1	72 (73.4)
2	19 (19.4)
3	6 (6.1)
4	1 (1)
BCLC Stage	
0	10 (10.2)
A	64 (65.3)
B	24 (24.5)
Type of surgery, n (%)	
Atypical resection	34 (34.7)
Segmentectomy	52 (53)
Sectionectomy	8 (8.1)
Hemi-hepatectomy	4 (4.1)
Extent of surgery, n (%)	
Major resection	4 (4.1)
Surgical approach, n (%)	
Laparoscopic	72 (73.4)
Robotic	8 (8.1)
Open	18 (18.5)
ASA score, n (%)	
2	37 (37.8)
3	61 (62.2)

IQR: inter-quartile range; BMI: body mass index; HBV: Hepatitis B virus; HCV: Hepatitis C virus; TACE: trans-arterial chemo-embolization; RFA: Radio-frequency ablation; ICG-R15: indocyanine green retention test at 15′; ASA: American Society of Anesthesiologists score.

**Table 2:** Analysis of (**A**) preoperative characteristics and (**B**) secondary outcomes according to HVPG measurement and clinically significant portal hypertension.

(**A**)			
	**No CSPH (84)**	**CSPH (14)**	***p*** **-Value**
Age in years, median (IQR)	72 (11)	73 (13.5)	0.98
Female gender, n (%)	12 (14.3)	4 (28.6)	0.18
BMI in kg/m^2^, median (IQR)	25.6 (5.1)	26.9 (6.6)	0.37
Etiology, n (%)			0.09
HBV	15 (17.9)	2 (14.3)	
HCV	27 (32.1)	4 (28.6)	
Alcoholic	8 (9.5)	2 (14.3)	
Metabolic	29 (34.5)	4 (28.6)	
Viral + metabolic	5 (6)	2 (7.2)	
Child–Turcotte–Pugh class			**<0.01**
Child A, n (%)	83 (98.8)	12 (85.7)	
Child B, n (%)	1 (1.2)	2 (14.3)	
Recurrent disease, n (%)	17 (20.2)	3 (21.4)	0.91
Previous treatments, n (%)	20 (23.8)	4 (28.6)	0.70
Previous TACE, n (%)	4 (4.9)	0 (0)	0.41
Previous RFA, n (%)	7 (8.3)	3 (21.4)	0.13
Previous liver surgery, n (%)	9 (10.7)	1 (7.1)	0.68
ICG-R15, median (IQR)	9.1 (9.4)	10.1 (22.4)	0.42
Liver stiffness, median in KPa (IQR)	9.8 (7.2)	20.7 (25.4)	**<0.01**
Esophageal varices, n (%)	8 (9.5)	5 (35.7)	**<0.01**
Splenomegaly, n (%)	9 (10.7)	9 (64.3)	**<0.01**
Platelets < 100 × 10^3^/mm^3^, n (%)	4 (4.8)	5 (35.7)	**<0.01**
HCC Size in mm, median (IQR)	37.5 (43)	29 (16)	0.33
HCC numbers, n (%)			0.35
1	64 (76.2)	8 (57.1)	
2	15 (17.9)	4 (28.6)	
3	4 (4.8)	2 (14.3)	
4	1 (1.2)	0 (0)	
BCLC Stage, n (%)			0.28
0	10 (11.9)	0 (0)	
A	55 (65.5)	9 (64.3)	
B	19 (22.6)	5 (35.7)	
(**B**)			
	**No CSPH (84)**	**CSPH (14)**	***p*** **-value**
Type of surgery, n (%)			0.40
Wedge resection	27 (32.2)	7 (50)	
Segmentectomy	46 (54.8)	6 (42.9)	
Sectionectomy	7 (8.3)	1 (7.1)	
Hemi-hepatectomy	4 (4.7)	0 (0)	
Extent of surgery, n (%)			0.39
Major resection	4 (4.7)	0 (0)	
ASA score, n (%)			0.38
2	33 (39.3)	3 (21.4)	
3	51 (60.7)	11 (78.6)	
Operation time, mean (SD)	389.5 (160.5)	343.7 (156.5)	0.42
EBL in ml, median (IQR)	300 (400)	300 (850)	0.96
Transfusions, n (%)	8 (13.1)	1 (9.1%)	0.71
Severe postoperative complications, n (%)	5 (8.2)	0 (0)	0.32
In-hospital mortality, n (%)	1 (1.2)	1 (7.1)	0.13
Length of stay, median (IQR)	5 (3)	7 (5)	0.36
90-day ascites, n (%)	2 (2.5)	3 (21.4)	**<0.01**
90 days decompensation, n (%)	1 (1.2)	3 (21.4)	**<0.01**
90-day mortality, n (%)	1 (1.2)	1 (7.1)	0.13

SD: standard deviation; IQR: inter-quartile range; BMI: body mass index; HBV: Hepatitis B virus; HCV: Hepatitis C virus; TACE: trans-arterial chemoembolization; ICG-R15: indocyanine green retention test at 15′; ASA: American Society of Anesthesiologists score; EBL: intraoperative estimated blood loss. In Bold statistically significant *p*-values.

## Data Availability

The datasets generated and analyzed during the current study are available from the corresponding author upon reasonable request.

## Supplementary Materials

The following supporting information can be downloaded at: https://www.mdpi.com/article/10.3390/cancers18142300/s1, Table S1: Additional exploratory subgroup analyses for predictive accuracy of Hepatic Venous Portal Gradient (HVPG), Indocyanine Green—Retention at 15 min (ICG-R15) and Liver Stiffness (LSM) for a composite endpoint defined as the development of PHLF, severe postoperative ascites, in-hospital mortality, severe postoperative complications.
