# Supplementary material for: Exploratory Evaluation of Hepatic Venous Pressure Gradient, Indocyanine Green Retention, and Liver Stiffness in Predicting Short-Term Outcomes After Liver Resection in Patients with Cirrhosis: A Multicenter Prospective Study
Source: Cancers (Basel). 2026 Jul 17;18(14):2300. doi: 10.3390/cancers18142300 (PMC13406228; doi:10.3390/cancers18142300)
Supplement: Supplementary file 1 [file cancers-18-02300-s001.zip › cancers-4353324-supplementary.pdf]

Stratification variable	Stratum	Predictor	AUC	95% CI	p_value
Major resection	0	HVPG	0.829	0.829 - 0.710	<0.001
Major resection	1	HVPG	0.643	0.643 - 0.364	0.315
Major resection	0	ICG-R15	0.597	0.597 - 0.342	0.456
Major resection	1	ICG-R15	0.439	0.439 - 0.132	0.695
Major resection	0	LSM	0.758	0.758 - 0.586	<0.001
Major resection	1	LSM	0.430	0.430 - 0.000	0.790
Minimally invasive	0	HVPG	0.667	0.395 - 0.938	0.229
Minimally invasive	1	HVPG	0.824	0.690 - 0.959	<0.001
Minimally invasive	0	ICG-R15	0.667	0.667 - 0.339	0.319
Minimally invasive	1	ICG-R15	0.508	0.508 - 0.225	0.957
Minimally invasive	0	LSM	0.544	0.543 - 0.189	0.812
Minimally invasive	1	LSM	0.751	0.751 - 0.547	0.01
Child Class	A	HVPG	0.787	0.661 - 0.912	<0.001
Child Class	A	ICG-R15	0.555	0.348 - 0.762	0.604
Child Class	A	LSM	0.685	0.496 - 0.873	0.055

Supplementary Material Table S1: Additional exploratory subgroup analyses for predictive accuracy of Hepatic Venous Portal Gradient (HVPG), Indocyanine Green—Retention at 15 min (ICG-R15) and Liver Stiffness (LSM) for a composite endpoint defined as the development of PHLF, severe postoperative ascites, in-hospital mortality, severe postoperative complications.
